# Moving Pictures

**Published:** 1913-08

**Authors:** 


### Moving Pictures

We shall have nothing to say as to the possibilities and actual accomplishments of the cinetograph in science, but venture to present an opinion as to the sociologic influence of ordinary, popular shows, rather at variance with the usual condemnations.

It is true that a great deal of time and money is spent at these shows, and by persons who cannot, apparently, afford it. But we live in an age when active efforts are made against an expenditure of half the income of a poor family for food, of a quarter for housing and a fifth for clothing. In other words, public sentiment, or rather public judgment, demands that everyone have education and enjoyment. On the whole, the moving picture show has solved better than any other the problem of cheap amusement. It has markedly threatened the high schedule of the regular theaters and has competed both with these and with various decidedly objectionable institutions for recreation, perhaps most of all with the cheapest directly and most expensive indirectly—loafing.

The moving picture show has been condemned as teaching immorality, using the term in a broad sense. Without pretending to a large experience, we have attended such shows in various cities covering nearly a third of the circumference of the globe and have never seen anything on the screen that would have been open to serious question on the theatric stage. It is true, however, that the imitative tendencies of children and childish adults have been stimulated by scenes of burglary, foolish antics, etc., but this potentiality of the moving pictures has been fairly well corrected by the system of censorship of films.

The mere availability of a place of retreat, more or less darkened, has led to some thefts and to more or less sexual immorality, truancy, etc. But similar charges may be made against almost everything which affords opportunity for relative release from restraint and supervision. They have been made against the bicycle, the automobile, the excursion boat, even the church and its associated activities. A considerable minority of the race cannot be trusted anywhere except in daylight, at a distance from other people and under the eye of a policeman. Instead of doing away with institutions which may possibly remove these restrictions, we believe in detecting this dependent class as soon as possible, though, of course, throwing safeguards around any particular spot of danger.

The moving picture theater has been condemned as unhygienic and as favoring the communication of disease. The same is true of almost every public gathering place and conveyance. A large part of our population is filthy and dangerous on account of its filth. At first thought it appears wise to eliminate whatever tends to bring this part of the population in contact with the decent and relatively safe part. On second thought, it is not improbable that the unwashed, who include many otherwise worthy persons, will gradually be educated by hearing others say "phew" at what must seem to them a normal atmosphere, and by being told in the newspapers that they stink. Anyhow, the moving picture show is not much worse than a political meeting, a popular concert or a near-end street car. Of course, we believe that ventilation, safety against fire, etc., should be inforced at these as well as at all similar gatherings.

The educative value of the moving picture show deserves some consideration, in spite of the tendency of recent years to paltry pantomime. In spite of these tedious and non-educative features which are no worse than for the majority of theatric presentations, there are enough travel scenes, pantomimes in foreign settings, current reviews and representations of historic events or of historic novels and plays, to have a decided value.

